# Isolation and characterization of the morphology, size and particle number of rainbow trout (Oncorhynchus mykiss) and zebrafish (Danio rerio) cell line derived large and small extracellular vesicles

**DOI:** 10.1007/s10695-023-01251-z

**Published:** 2023-10-23

**Authors:** Csilla Pelyhe, Joachim Sturve

**Affiliations:** Department of Biological and Environmental Sciences, University of Gothenburg, Gothenburg, Sweden

**Keywords:** Extracellular vesicles, Exosome, Vesicle, Rainbow trout, Zebrafish, Piscine cell line

## Abstract

Extracellular vesicles (EVs) are 50–1,000 nm lipid bilayer-bound vesicles, released into the extracellular environment by various cell types for intercellular communication purposes. The quantitative and qualitative characteristics of EVs can be affected by stress and pathological conditions. The majority of extracellular vesicle (EV) studies have been performed on mammalian cell lines or bodily fluids. EVs have been previously described from bodily fluids like plasma, serum or mucus in different fish species, however the available knowledge of fish cell line derived EVs is limited and in the vast majority of studies, the overall focus is on small EVs (< 200 nm). We isolated large and small extracellular vesicles from zebrafish (*Danio rerio*) liver (ZFL), rainbow trout (*Oncorhynchus mykiss*) liver (RTL-W1), gill (RTgill-W1) and intestinal epithelial (RTgutGC) cell lines using stepwise centrifugation and characterized the size and morphology of EVs. Here we demonstrated that large and small extracellular vesicles can be successfully isolated using stepwise centrifugation from the serum-free medium of the selected piscine cell lines after a 24-h incubation period. The size distribution of large and small EVs isolated from the piscine cell lines suggest that large and small EV groups show high diversity in size ranges, containing heterogenous subpopulations in sizes, and the results highly depend on the applied method and whether filtration steps were included following the isolation. The spherical morphology of EVs was verified by transmission electron microscopy.

## Introduction 

Extracellular vesicles (EVs) are lipid bilayer-bound vesicles of approximately 50–1,000 nm in diameter, released into the extracellular environment by various cell types for intercellular communication purposes (Raposo and Stoorvogel 2013). Based on their morphologies, biogenesis, or contents, EVs are classified into three main categories: apoptotic bodies, microvesicles and exosomes (Mashouri et al. 2019). Microvesicles are derived by budding or blebbing off the plasma membrane, and they have a diameter of approximately 100–1,000 nm. While exosomes originate from the multivesicular bodies and are released through exocytosis with a typical size of approximately 30–200 nm (Crescitelli et al. 2013; Raposo and Stoorvogel 2013) . However, recent studies suggest that these two subgroups are composed of several EV subpopulations (Kowal et al. 2016; Willms et al. 2016; Lässer et al. 2017; Zabeo et al. 2017) and most recently the nomenclature was suggested as small EVs (< 100 nm) and large EVs (100–1000 nm) (Crescitelli et al. 2021). EVs are shed continuously and are present in most bodily fluids. They transport diverse molecules including proteins, enzymes, genetic material, long non-coding RNAs and microRNAs, derived from the cells of origin as information cargo (Raposo and Stahl 2019). The number and cargo of the released EVs can be affected by stress or diseases (Raposo and Stoorvogel 2013), resulting in the potential of usage of EVs as biomarkers. The cargo of EVs depends on the parent cell and reflects its responses to stress (Palviainen et al. 2019), while the uptake of EVs is leading to functional changes in the recipient cells (O'Brien et al. 2020). Apoptotic bodies range from 50 to 5,000 nm in diameter, are produced from cells undergoing programmed cell death, and carry nuclear fragments and cellular organelles such as mitochondria and endoplasmic reticulum as a result of apoptosis (Kakarla et al. 2020).

EV research has been exponentially expanding in the past decade in the human medical field, investigating EVs as biomarkers or even as tools in potential therapy, however, less is known about EVs in other species (Zhao et al. 2022). According to a recent review, extracellular vesicles were studied only in 61 aquatic species, including less than 10 fish species (Zhao et al. 2022). EVs have been reported in different fish species from seminal plasma, blood plasma, serum, epithelial mucus (Zhao et al. 2022), including Atlantic cod (*Gadus morhua L.*) (Lange et al. 2019; Magnadóttir et al. 2019; Magnadóttir et al. 2020), Atlantic salmon (*Salmo salar*) (Iliev et al. 2010; Lagos et al. 2017; Iliev et al. 2018; Smith et al. 2020), Rainbow trout (*Oncorhynchus mykiss*) (Faught et al. 2017; Cadonic et al. 2020) and Zebrafish (*Danio Rerio*) (Ohgo et al. 2020; Scott et al. 2021; Kobayashi-Sun et al. 2020), however, the vast majority of these studies focus only on small EVs, excluding large EVs.

Zebrafish (*Danio rerio*) and rainbow trout (*Oncorhynchus mykiss*) are commonly used models in aquaculture in many different areas (Braunbeck et al. 1992; Liu et al. 2013; Schartl 2014). However, publications presenting EVs in rainbow trout are scarce (Faught et al. 2017; Cadonic et al. 2020). Zebrafish is widely used in the human medical field as a model (Goldsmith 2004) and in some studies it is used as a recipient of EVs of mammalian-origin (Zhao et al. 2022). In addition, specific EV-marker-labelled models of zebrafish (Scott et al. 2021) have been published to demonstrate and describe the biogenesis and uptake of EVs in vivo and EVs have been reported from fin blastema (Ohgo et al. 2020) and osteoblast-derived extracellular vesicles in zebrafish (Kobayashi-Sun et al 2020). However there is no available data on isolation and characterization of EVs in vitro from zebrafish or rainbow trout cell lines.

The aim of this study was to isolate and characterize large and small extracellular vesicles using piscine cell lines of different species and/or tissues. Namely, we aimed to isolate and characterize EVs from zebrafish liver- (ZFL), rainbow trout liver-(RTL-W1), gill (RTgill-W1) and intestinal epithelial cell line (RTgutGC) and characterize the number, size and morphology of large and small EVs.

## Materials and methods

### Cell cultures

Piscine derived cell lines were maintained as described earlier (Thit et al. 2017; Lammel and Sturve 2018).

The rainbow trout (*Oncorhynchus mykiss*) liver cell line (RTL-W1) (Lee et al. 1993), rainbow trout intestinal epithelial derived cell line (RTgutGC) (Kawano et al. 2011) and rainbow trout gill cell line (RTgill-W1) (Bols et al. 1994) were cultured in T75 cell culture flasks (TC Flask T75, Sarstedt) in phenol red-free Leibovitz’s L-15 Medium (Gibco, Thermo Fisher Scientific) supplemented with 5% fetal bovine serum (FBS) (Gibco). Rainbow trout cell line flasks were incubated at 19 °C and split in ratios of 1:2 or 1:3 when reaching confluence using 0.2 g/l ethylenediaminetetraacetic acid (EDTA)/phosphate buffered saline (PBS) and 0.25% trypsin–EDTA solution (Gibco).

The zebrafish (*Danio rerio*) liver cell line (ZFL; CRL-2643) was purchased from LGC (UK). Cells were maintained in T75 cell culture flasks (TC Flask T75, Sarstedt) in phenol red-free Leibovitz’s L-15 Medium (Gibco, Thermo Fisher Scientific) supplemented with 5% fetal bovine serum (FBS) (Gibco). The flasks were incubated at 27 °C and split in ratios of 1:5 or 1:10 when reaching confluence using 0.2 g/l ethylenediaminetetraacetic acid (EDTA)/phosphate buffered saline (PBS) and 0.25% trypsin–EDTA solution (Gibco).

## Extracellular vesicle isolation

**Fig. 1:**
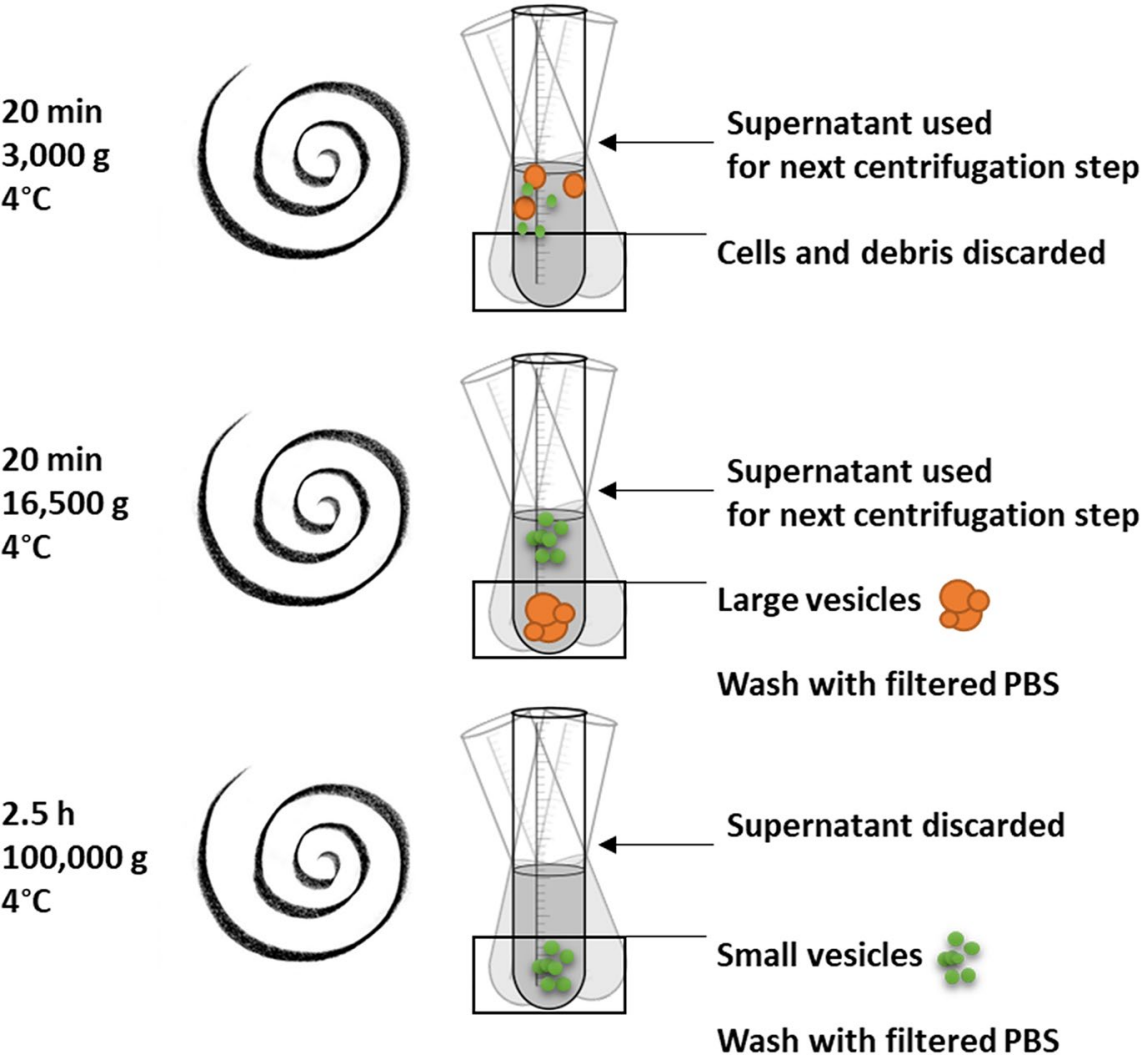
Schematic figure of isolation of large and small EVs. EV isolates were prepared from 8 mL serum-free medium after a 24-h incubation on each cell line in a T75 flaks with full confluency. Medium was collected and centrifuged at 3000 g for 20 min at 4 °C, to ensure the removal of aggregates and apoptotic bodies. The supernatants containing the EVs were collected to a clean tube and centrifuged at 16,500 g to pellet large EVs. The supernatant was collected to clean tubes and ultracentrifuged at 100,000 g for 2.5 h at 4 °C to pellet small EVs. Both large- and small EV pellets were washed in 0.2 micron filtered PBS and recentrifuged as described earlier. The EV-enriched pellets were resuspended in 100 µL filtered PBS and used as a fresh sample or stored at -80 °C for further analysis

Full confluent T75 flasks of cells were incubated in serum-free phenol red-free Leibovitz’s L-15 Medium (Gibco, Thermo Fisher Scientific) for 24 h, then EVs were isolated from the collected medium by stepwise centrifugation. This method was adapted from mammalian cell cultures (Crescitelli et al. 2021) and modified to piscine cell lines according to the guidelines of the International Society for Extracellular Vesicles (ISEV) (Théry et al. 2018). EV isolates were prepared from 8 mL medium, which were centrifuged at 3000 g for 20 min at 4 °C (Beckman Coulter Avanti J-26XP, JA-21 Fixed-Angle Rotor), to ensure the removal of cells and aggregates. The supernatants containing the EVs were collected to a clean tube and centrifuged at 16,500 g for 20 min at 4 °C to pellet large EVs (Beckman Coulter Avanti J-26XP, JA-21 Fixed-Angle Rotor). The supernatant was collected to clean tubes and ultracentrifuged at 100,000 g for 2.5 h at 4 °C to pellet small EVs (Beckman Coulter L8-70 M, Type 50.4 Ti Fixed-Angle Titanium Rotor). Both large and small EV pellets were washed in 0.2 µm filtered PBS and re-centrifuged as described above. The EV-enriched pellets were resuspended in 100 µL 0.2 µm filtered PBS and used as a fresh sample or stored at -80 °C for further analysis (Fig. 1).

## Transmission Electron Microscopy (TEM)

The morphology of EVs was characterized by Transmission Electron Microscopy (TEM) as follows: fish cell line-derived EVs were used for morphological analysis using TEM according to previously described methods (Crescitelli et al. 2021). In brief, freshly isolated large and small EV pellets were resuspended in 0.2 µm filtered PBS, 20 µL sample was placed on a piece of parafilm, Formvar/carbon-coated hexagon mesh grids were glow discharged in GloQube Plus Glow Discharge system (Quorum, cat. no. 025235) and placed on top of the sample for 10 min, samples were fixed at room temperature for 5 min in 2.5% glutaraldehyde (vol/vol) in Milli-Q H_2_O (8% (vol/vol) water solution; Electron Microscopy Sciences, cat. no. 16020). For the staining of EVs, 2% Uranyl Acetate (wt/vol) in Milli-Q H2O was used for 1.5 min (Merck, cat. no. 8473). EV imaging was performed using Thermo Scientific™ Talos L120C TEM (Thermo Fisher Scientific Co., Ltd., USA) transmission electron microscope, 4 k × 4 k Ceta CMOS camera (Thermo Fisher Scientific Co., Ltd., USA) and the MAPS™ software (Thermo Fisher Scientific Co., Ltd., USA). Images were captured at low magnification (20,000–40,000 ×) and high magnification (60,000–80,000 ×).

## Tunable Resistive Pulse Sensing (TRPS) analysis

Large and small EV samples were analyzed by Tunable Resistive Pulse Sensing (TRPS) using a qNano gold instrument (IZON Sciences Ltd.) as described previously (Szabó et al. 2014; Osteikoetxea et al. 2015; Vukman et al. 2020) and optimized to our experiments. Briefly, NP400 (analysis range: 185–1100 nm particles) and NP2000 nanopore membrane (analysis range: 935–5700 nm particles) were used to measure the large EV samples and NP100 (analysis range: 50–330 nm) nanopore membrane was used to measure the small EV samples. All samples were diluted 1:10 in 0.2 µm filtered PBS. Samples were vortexed and filtered with 1.0 µm or 0.2 µm filters, depending on large or small EV samples were analyzed, to remove larger particles prior to the measurement preventing pore clogging. We counted at least 500 events/sample, following the distributor’s recommendation. Calibration was performed using calibration beads with a defined concentration, provided by the manufacturer (IZON). Results were evaluated using the IZON Control Suite 3.2 software. 1.0 µm and 0.2 µm filtered PBS samples were also measured and used to subtract background noise in the calculation. Data was grouped and merged as 200–300 nm, 301–400 nm, 401–500 nm, 501–600 nm, 601–700 nm for NP400 measurements and 500–600 nm, 601–700 nm, 701–800 nm, 801–900 nm, 901–1000 nm, 1001–1200 nm, 1201–1300 nm, 1301–1400 nm, 1401–1500 nm, 1501–1600 nm, 1601–1700 nm, 1701–1800 nm, 1801–1900 nm,1901–2000 nm and 2001–2100 nm for NP2000 measurements for plotting size distribution of large EVs with the average and SD of 3 replicates (n = 3), then the NP400 and NP2000 data was merged to one size distribution diagram for each EV group in each cell line. Data was plotted from 250 nm for large EV samples, since the NP400 did not measure 0-250 nm size range. Data was grouped and merged as 0–50 nm, 51–100 nm, 101–150 nm, 151–200 nm and 201–250 nm for plotting size distribution of small EVs with the average and SD of 3 replicates (n = 3) for each EV group in each cell line. Small EV samples were not measured with NP400 and NP2000 since the samples were filtered with 0.2 µm filters prior to measurement to remove aggregates, while large EVs were not measured with NP100 to avoid pore clogging.

## Dynamic light scattering (DLS) analysis

Dynamic light scattering (DLS) analysis was performed on a Zetasizer Nano-ZS apparatus (Malvern Instruments Ltd., Malvern, UK) using disposable polystyrene micro cuvettes (VWR International AB, Göteborg, Sweden). Both large and small EV samples (n = 3) were diluted in 0.2 µm filtered PBS. Samples were vortexed and filtered with 1.0 µm or 0.2 µm filters, depending on large or small EV samples were analyzed, to remove aggregates. The attenuation level and optimum measurement position was automatically determined by the instrument. The measurement temperature was set to 20 °C. The general purpose (normal resolution) analysis model was selected for result calculation. The software used for analysis and visualization of DLS data was Zetasizer software version 7.11 (Malvern Instruments Ltd.) where size distribution by intensity (intensity percent) was selected to demonstrate the results. 0.2 µm filtered PBS sample was also measured and used to subtract background noise in the calculation. DLS analysis data is displayed 0- 2,000 nm for large EV and 0–1,000 nm for small EV. The averages and SDs of 3 replicates (n = 3) were plotted as size distribution diagrams for each EV group in each cell line. Data > 2000 nm was not shown for large EV, since the samples were filtered with 1.0 µm filters prior to measurement to remove aggregates.

## Results

### Piscine cell line derived large and small extracellular vesicles are highly heterogeneous in size

Size, particle number and morphology of piscine cell line-derived EVs were characterized by TRPS, DLS and TEM.

### Piscine cell line derived large and small EVs showed distinguished profile in size distribution and high heterogeneity in particle number in Tunable Resistive Pulse Sensing (TRPS) analysis

**Fig. 2:**
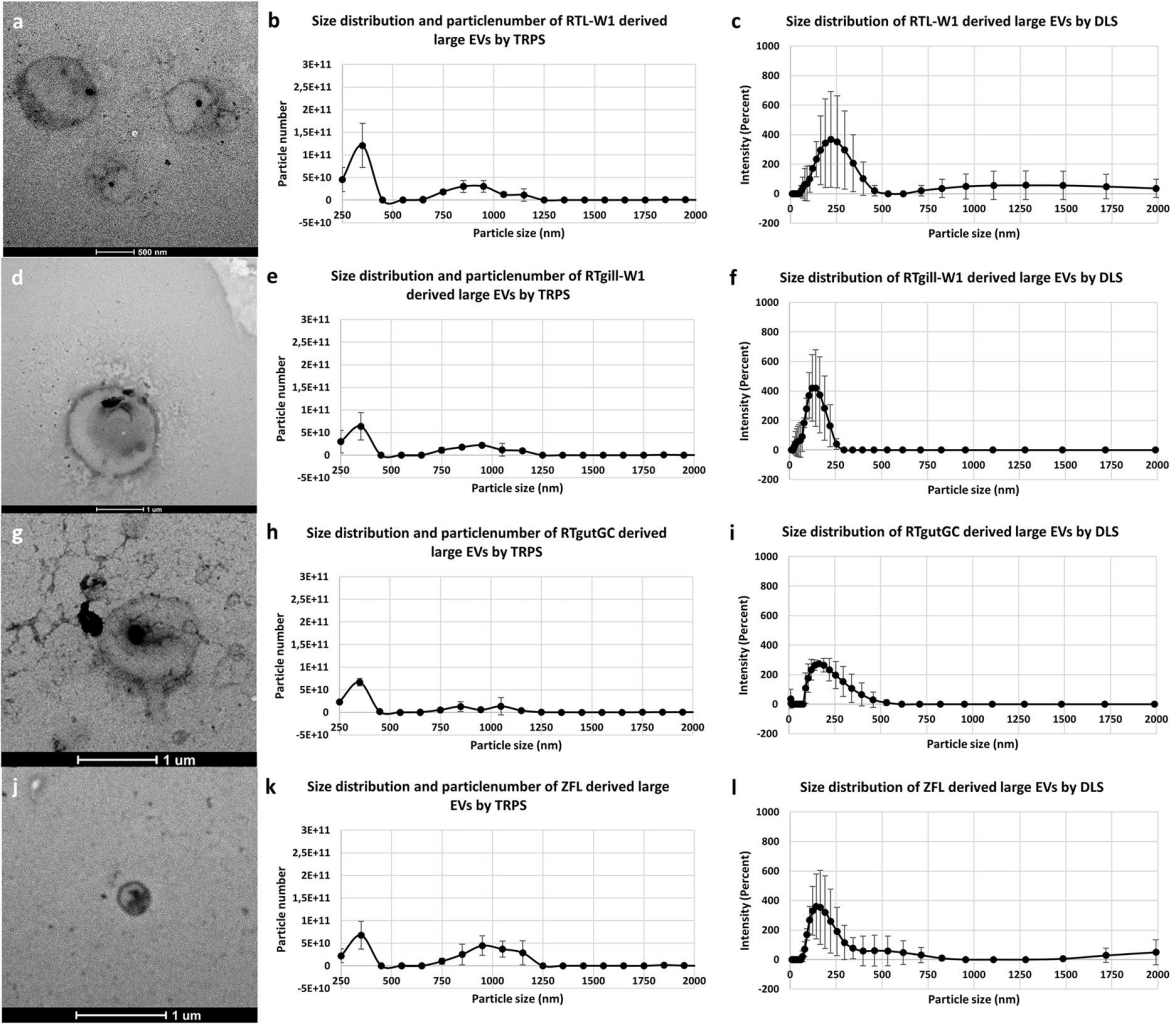
Representative images of the size distribution and morphology of large extracellular vesicles isolated from piscine cell lines. TEM images of the RTL-W1 (a); RTgill-W1 (d); RTgutGC (g) and ZFL (j) cell line derived large EVs. Size distribution and particle number of RTL-W1 (b); RTgill-W1 (e); RTgutGC (h) and ZFL (k) cell line derived large EVs measured by TRPS. Size distribution of RTL-W1 (c); RTgill-W1 (f); RTGC (i) and ZFL (l) cell line derived large EVs measured by DLS

RTL-W1 derived large EVs showed a dominant peak between 250 and 350 nm and a second peak at 650 and 1250 nm. We measured the highest particle concentration 350 nm particle size, where 1.21E + 11 ± 4.89E + 10 particles/mL concentration was measured (Fig. 2b). RTgill-W1 derived large EVs measured a dominant peak between 250 and 450 nm, and a second peak at 650 and 1150 nm was showed. We measured the highest particle concentration at 350 nm particle size, where 6.39E + 10 ± 3.1E + 10 particles/mL concentration was measured (Fig. 2e). In RTgutGC derived large EVs a dominant peak was measured between 250 and 450 nm, and a second peak at 650 and 1250 nm was showed. We measured the highest particle concentration at 350 nm particle size, where 6.7E + 10 ± 8.2E + 09 particles/mL concentration was measured (Fig. 2h). ZFL derived large EVs showed a dominant peak between 250 and 350 nm and a second peak at 650 and 1150 nm was showed. We measured the highest particle concentration at 350 nm particle size, where 6.79E + 10 ± 3.09E + 10 particles/mL concentration was measured (Fig. 2k).

**Fig. 3:**
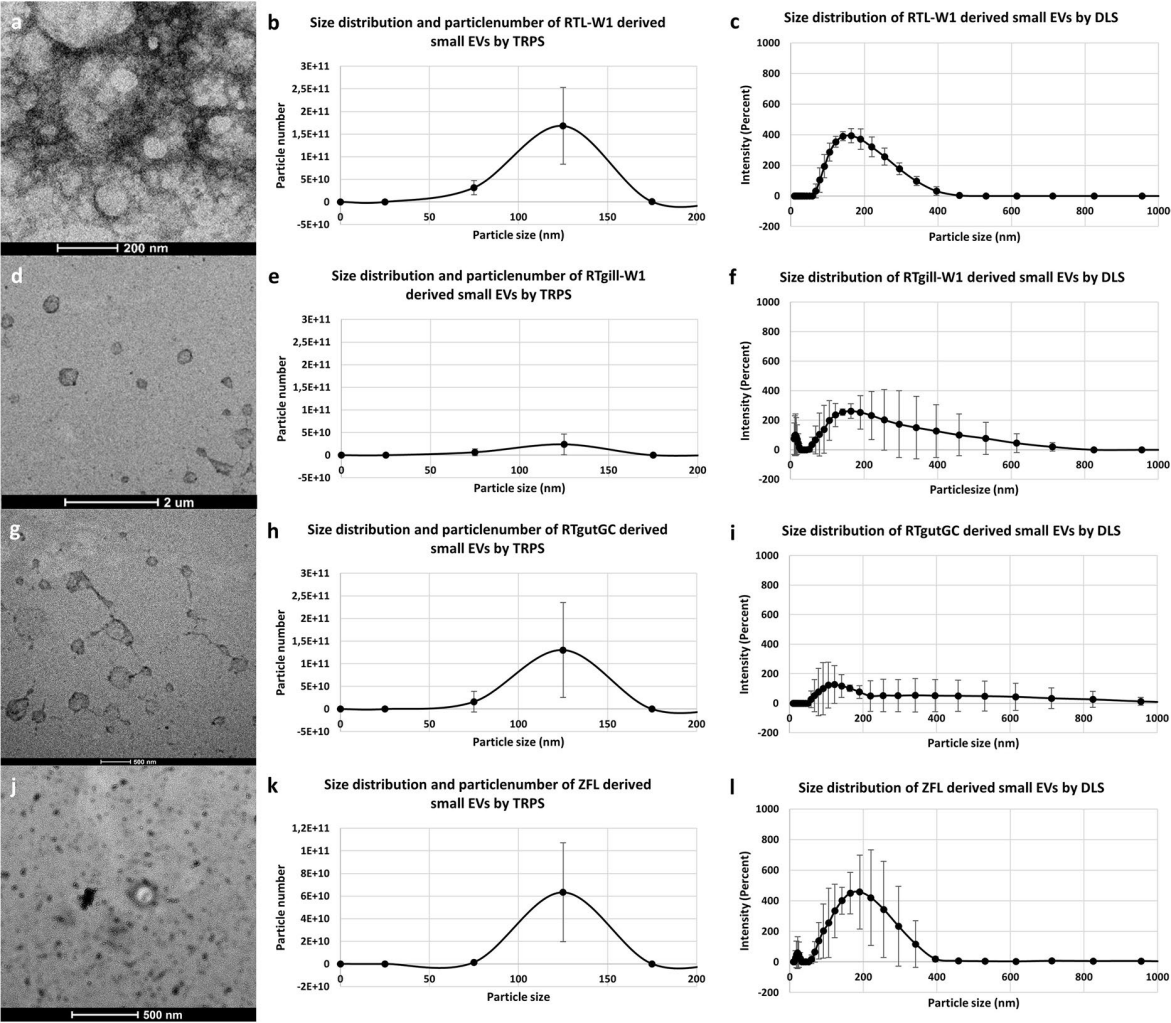
Representative images of the size distribution and morphology of small extracellular vesicles isolated from piscine cell lines. TEM images of the RTL-W1 (a); RTgill-W1 (d); RTGC (g) and ZFL (j) cell line derived small EVs. Size distribution and particle number of RTL-W1 (b); RTgill-W1 (e), RTgutGC (h) and ZFL (k) cell line derived small EVs measured by TRPS. Size distribution of RTL-W1 (c); RTgill-W1 (f); RTgutGC (i) and ZFL (l) cell line derived small EVs measured by DLS

RTL-W1 derived small EVs measured a single peak between 75 and 225 nm. We measured the highest particle concentration at 125 nm particle size, where 1.68E + 11 ± 8.45E + 10 particles/mL concentration was measured (Fig. 3b). In RTgill-W1 derived small EVs a single peak was observed between 75 and 225 nm. We measured the highest particle concentration at 125 nm particle size, where 3.37E + 10 ± 52.26E + 10 particles/mL concentration was measured (Fig. 3e). RTgutGC derived small EVs showed a single peak between 75 and 225 nm. We measured the highest particle concentration at 125 nm particle size, where 1.3E + 11 ± 1.05E + 11 particles/mL concentration was measured (Fig. 3h). ZFL derived small EVs measured a single peak between 75 and 225 nm. We measured the highest particle concentration at 125 nm particle size, where 6.34E + 10 ± 4.37E + 10 particles/mL concentration was measured (Fig. 3k). TRPS data is summarized in Table 1.

**Table 1:** Summary of size and particle number of EVs with sample volume used for EV isolation in different fish species

Species and EV source	Size distribution by NTA or nano-flowcytometry (peaks) (nm)	Size distribution (peak) by DLS (nm)	Size distribution (peak) by TRPS (nm)	Particle number (particles/mL) by NTA	Particle number (particles/mL) by TRPS	Sample volume (μl)	Reference
Rainbow trout gut (large EV) RTgutGC supernatant		91–531 (164)	250–1250 (350)		6.7E + 10 ± 8.2E + 09	8,000	present data
Rainbow trout gut (small EV) RTgutGC supernatant		58–1106 (122.4)	75–225 (125)		1.3E + 11 ± 1.05E + 11		
Rainbow trout gill (large EV) RTgill-W1 supernatant		21–255 (164)	250–1150 (350)		6.39E + 10 ± 3.1E + 10		
Rainbow trout gill (small EV) RTgill-W1 supernatant		10–712 (164)	75–225 (125)		3.37E + 10 ± 52.26E + 10		
Rainbow trout liver (large EV) RTL-W1 supernatant		58–2,000 (220)	250–1250 (350)		1.21E + 11 ± 4.89E + 10		
Rainbow trout liver (small EV) RTL-W1 supernatant		68–458 (164)	75–225 (125)		1.68E + 11 ± 8.45E + 10		
Zebrafish cell line liver (large EV) ZFL supernatant		68–2,000 (141.8)	250–1150 (350)		6.79E + 10 ± 3.09E + 10		
Zebrafish cell line liver (small EV) ZFL supernatant		58–955 (190.1)	75–225 (125)		6.34E + 10 ± 4.37E + 10		
Atlantic cod mucus	30–500 (142)			5.8e + 9		200	Magnadóttir et al., 2019
Atlantic cod mucus and plasma	30–400 (120)			6.5e + 8		200–250	Lange et al. 2019
Atlantic cod plasma	30–500 (160)			5.0–7.0e + 10		250	Magnadóttir et al. 2020
Atlantic salmon plasma	60–250 (124/106)			6.62e + 8—7.08e + 8		3,000	Muñoz et al., 2022
Salmon headkidney leukocytes supernatant	107.6–137			2.0–2.2E + 8		2,000	Smith et al. 2020
Coho salmon plasma	56–278 (190)			1.6E + 6–1.1E + 7		200	Leiva et al. 2021
Chinese tongue sole plasma	20–120 (95)			1.80e + 9—2.71e + 9		40,000	Sun et al. 2017
Chinese tongue sole plasma	100–400 (142)			2.40 ± 0.08e + 9		na	Zhao et al. 2021
Chinese tongue sole plasma	50–200*			5.28e + 8—2.71e + 9		na	Zhu et al. 2022

### Piscine cell line derived large and small EVs showed similar profile in size distribution in Dynamic light scattering (DLS) analysis

RTL-W1 derived large EVs (Fig. 2c) showed a main peak between 58.77 and 459 nm and a second minor peak between 712 and 3000 nm. The highest intensity was measured at 220.2 nm (367.8% ± 325.1). In RTgill-W1 derived large EVs a single peak was observed between 21.04 and 255 nm in DLS analysis (Fig. 2f), where the highest intensity was measured at 164.2 nm (373.3% ± 257.4). RTgutGC derived large EVs also measured a single peak between 91.3 and 531.2 nm (Fig. 2i). The highest intensity was measured at 190.1 nm (263.3% ± 45.8). While ZFL derived large EVs also showed a single peak between 68.06 and 825 nm, where the highest intensity was measured at 164.2 nm (353.3% ± 251.1) (Fig. 3l).

In RTL-W1 derived small EVs a single peak was observed between 59 and 459 nm (Fig. 3c), where the highest intensity was measured at 164.2 nm (393.3% ± 46.2). While RTgill-W1 derived small EVs measured a minor peak at 10–24 nm and a major peak between 50.75 and 712.4 nm. The highest intensity was measured at 164.2 nm (261.7% ± 49.5) (Fig. 3f). RTgutGC derived small EVs showed a single main peak between 58.77 and 1106 nm. The highest intensity was measured at 122.4 nm (126.7% ± 127.3) (Fig. 3i). ZFL derived small EVs showed a single main peak between 58.77 and 955.4 nm. The highest intensity was measured at 190.1 nm (456.7% ± 241.3) (Fig. 3l). DLS data is summarized in Table 1.

### Piscine cell line derived large and small EVs showed lipid bilayer-bound structures in various sizes in Transmission Electron Microscopy (TEM)

Morphological analysis using TEM confirmed the polydispersed EV population in every studied piscine cell line. Both large and small EV samples showed nanosized particles of very heterogenous sizes. Spherical structured vesicles are visible with sizes approximately 200–2000 nm RTL large EV samples (Fig. 2a); RTgill-W1 large EV samples (Fig. 2d); RTgutGC large EV samples (Fig. 2g) and ZFL large EV samples (Fig. 2j), while small EVs with approximately smaller size than 500 nm in diameter were observed in RTL small EV samples (Fig. 3a); RTgill-W1 small EV samples (Fig. 3d); RTgutGC samples (Fig. 3g) and ZFL samples (Fig. 3j).

## Discussion

In the present study we characterized the number, size and morphology of rainbow trout (*Oncorhynchus mykiss*) and zebrafish (*Danio rerio*) cell line derived extracellular vesicles (EVs), namely large and small EVs of rainbow trout liver (RTL-W1), gill (RTgill-W1), intestinal epithelium (RTgutGC) and zebrafish liver (ZFL) cell lines. Morphology and size distribution of EVs isolated from the piscine cell lines were characterized according to the Minimal Information for Studies of Extracellular Vesicles (2018) (MISEV 2018) guidelines (Théry et al. 2018), using DLS, TRPS and TEM. Here we demonstrate that stepwise centrifugation combined with filtration is a suitable method to isolate large and small EVs from the selected piscine cell lines, using the serum-free medium as a source for isolation after a 24-h incubation period. In this study we used 3000 g for 20 min to remove cell debris, 16,500 g for 20 min combined with 1 µm filter to isolate large vesicles, while 100,000 g for 2.5 h combined with 0.2 µm filer to isolate small vesicles. We characterized the large and small EVs with spherical or saucer-like morphology by TEM.

For large EVs the highest particle concentration was at 350 nm for every piscine cell line, where the highest particle number was 1.21E + 11 ± 4.89E + 10 particles/mL for RTL-W1, 6.39E + 10 ± 3.1E + 10 particles/mL for RTgill-W1, 6.7E + 10 ± 8.2E + 09 particles/mL for RTgutGC and 6.79E + 10 ± 3.09E + 10 particles/mL for ZFL. For small EVs the highest particle concentration was at 125 nm for every piscine cell line, where the highest particle number was 1.68E + 11 ± 8.45E + 10 particles/mL for RTL-W1, 3.37E + 10 ± 52.26E + 10 particles/mL for RTgill-W1, 1.3E + 11 ± 1.05E + 11 particles/mL for RTgutGC and 6.34E + 10 ± 4.37E + 10 particles/mL for ZFL. While in DLS analysis the highest intensity was measured at 220.2 nm for RTL-W1, 164.2 nm for RTgill-W1, 190.1 nm for RTgutGC and 164.2 nm for ZFL derived large EVs, and 164.2 nm for RTL-W1, 164.2 nm for RTgill-W1, 122.4 nm for RTgutGC and 190.1 nm for ZFL derived small EVs (Table 1). These datasets show that there are many different subpopulations in large and small EVs, even if we study only their size, and the results will depend on the sensitivity and accuracy of the applied method (Anderson et al. 2013).

EVs are present in most bodily fluids for cell–cell communication purposes. The quality and quantity of released EVs can be affected by stress or diseases (Raposo and Stoorvogel 2013) resulting in the potential of using EVs as biomarkers.

Currently the research of EVs in aquatic biology is limited. According to a recent review, extracellular vesicles were studied in less than 10 fish species with a total of 35 scientific papers (Zhao et al. 2022) showing high variety in the applied methods in EV isolation and characterization. Indeed, the vast majority of the studies focus on the small EVs (< 100 nm) and use 0.1 µm filtration during the isolation, excluding the larger vesicle populations from their studies, despite that EVs are defined between 50–1,000 nm size distribution (Raposo and Stoorvogel 2013).

Only a few publications are available presenting EVs in rainbow trout (Faught et al. 2017; Cadonic et al. 2020), where small EVs were studied as response to heat stress in vivo isolated from blood plasma and in vitro from primary hepatocytes (Faught et al. 2017). Faught et al. (2017) used the following steps for isolation: 1200 g for 20 min, 10,000 g for 30 min, 150,000 g 120 min to pellet the small EVs. While, Cadonic et al. (2020) used a combination of centrifugation for 12,000 g for 1 h and filtration (0.22 µm) for isolation of small vesicles from rainbow trout plasma and analyzed the miRNA cargo of EVs in air-stress response of rainbow trout in vivo. Cadonic et al. (2020) and Faught et al. (2017) characterized the size of small EVs based on the TEM photos and found 50-100 nm vesicles with a spherical morphology. HSP70 was used as EV marker in Western blot by Faught et al. (2017), while Cadonic et al., (2020) did not include canonical EV markers in Western blot analysis in their study.

Reports describing EVs in other bony fish species is also limited (Zhao et al. 2022), including Atlantic cod (*Gadus morhua L.*) (Lange et al. 2019; Magnadóttir et al. 2019; Magnadóttir et al. 2020), Atlantic salmon (*Salmo salar*) (Iliev et al. 2010; Lagos et al. 2017; Iliev et al. 2018; Smith et al. 2020, Leiva et al. 2021; and Muñoz et al. 2022) and Zebrafish (*Danio Rerio*) (Ohgo et al. 2020; Scott et al. 2021; Kobayashi-Sun et al. 2020).

Lange et al. (2019) isolated EVs from Atlantic cod sera and mucus using the following steps: 4,000 g for 30 min and 100,000 g for 1 h and studied the deiminated forms of C4-like protein, EVs were analyzed with NTA and TEM, however, these data were published in supplementary and the study focuses on C4 protein. The methodology description does not contain filtration. Magnadóttir et al. 2019 and 2020) reported polydispersed populations of EVs derived from Atlantic cod mucus and plasma using the following steps: 4,000 g for 30 min and 100,000 g for 1 h. The methodology description does not contain filtration. The size range of EVs was reported 30–500 nm, 5.8e + 9 particles/mL concentration of mucus origin (2019) and 30-500 nm size range with 5–7.0e + 10 particles/mL concentration of plasma origin (2020) measured by NTA and TEM. Atlantic cod mucus and plasma derived EVs were analyzed for immune factors and protein cargo, while serum derived EVs were studied for miRNA cargo (Magnadóttir et al. 2019 and 2020). Lange et al. (2019) and Magnadóttir et al. (2019 and 2020) reported a saucer-like morphology of the EVs in the TEM analysis. They also used CD63 and Flotillin-1 as EV markers in Western blot analysis (Magnadóttir et al. 2019 and 2020; Lange et al. 2019).

Iliev et al. (2010) isolated EVs from salmon plasma using the following steps: 500 g 5 min, 1200 g 20 min, 10,000 g 30 min and 115,000 g for 1 h. The methodology description does not contain filtration. They reported that following stimulation with bacterial lipopolysaccharide and DNA, antigen presenting cells isolated from salmon head kidney degranulate and secrete MHC-II-β containing vesicles with characteristics of exosomes. TEM was used to describe the size of EVs as up to 100 nm in diameter and a saucer-like morphology. Iliev et al. (2018) also isolated EVs from primary cultures of head kidney leukocytes from Atlantic salmon (*Salmo salar*); and ASK (*Salmo salar*) and CHSE-214 (O*ncorhynchus tshawytscha*) cell lines and found that treatments like phosphorothioate oligonucleotides and genomic DNA or heparin induce secretion of exosomes. They were using 500 g 10 min, 1500 g 15 min, 10 000 g 40 min, then 0.2 μm filtration and 114 000 g 2 h and gradient ultracentrifugation 114 000 g 3 h. EVs were described with saucer like morphology. In this study Iliev et al. (2018) also used HEK293T cells (human embryonic kidney cells) where 1500 g 15 min was followed by ultracentrifugation at 114,000 g to isolate exosomes and larger EVs together. They also used EV markers, MHC-II (Iliev et al. 2010) or Alix and Flotillin-1 in Western blot analysis (Iliev et al. 2018).

Lagos et al. (2017) isolated EVs from serum of *Piscirickettsia Salmonis* infected Atlantic Salmon using 10,000 g 30 min centrifugation and exoEasy Maxi Kit and analyzed the protein cargo of the EVs of healthy and infected fish origin. They described salmon plasma derived small EVs of average diameter of 230 nm–300 nm using NTA and flow cytometry with a saucer-like morphology in TEM, while they did not include EV markers in Western blot analysis in their study.

While, Leiva et al. (2021) isolated EVs from *Piscirickettsia salmonis-*infected Coho Salmon and analyzed the miRNA cargo of EVs using a combination of filtration (0.22 µm), precipitation and 12,000 g for 1 h centrifugation for isolation of vesicles. They described small EVs 56–278 nm with 1.6E + 6–1.1E + 7 particles/mL concentration measured by NTA with spherical shaped morphology by TEM, while they did not include EV markers in Western blot analysis in their study.

Muñoz et al. (2022) described plasma derived EVs from *Piscirickettsia salmonis*-infected Atlantic salmon using the combination of: 1,500 g 10 min, 10,000 g 10 min centrifuge steps with qEV size exclusion columns separating large EVs, exosomes and serum proteins and analyzed RNA and protein cargo of small EVs. EVs were isolated in a range of 60-250 nm with 6.62E + 8—7.08E + 8 particles/mL concentration measured by NTA, with spherical morphology at a size range of 50–125 nm by TEM, and using an EV marker MHC-II in western blot.

Smith et al. (2020) described salmon head-kidney leukocytes derived small EVs isolated with the combination of Vn96 peptide precipitation and 17,000 g for 15 min centrifugation in a range of 107.6–137.6 nm and 2.0–2.2E + 8 particles/mL concentration measured by Nanoparticle Tracking Analysis (NTA) and with a mixed morphology of spherical and saucer-like particles by TEM. They also used HSP90 as EV marker in Western blot.

In zebrafish the available publications are of different concept since the amount of vesicles is limited, however, Scott et al. (2021) developed a transgenic zebrafish model with labelled EVs for in vivo imaging and also analyzed the EVs from dissociated cells of adult ventricules and whole larvae zebrafish with a combination of using 300 g 10 min, 1200 g 10 min, 10 000 g 30 min, 1.0 μm filtration, 118 000 g (1 h 54 min) and sucrose density gradient ultracentrifugation 179 500 g (20 h). They described the morphology of EVs as round or saucer-like particles using TEM with a size range of 20 nm-820 nm using NTA and DLS and they also used EV markers, Alix and Syntenin in Dot blot analysis. Ohgo et al. (2020) also developed an in vivo transgenic zebrafish model with labelled EVs and studied the process of fin regeneration and observed CD63 and CD9 expression using in vivo electroporation. While Kobayashi-Sun et al., (2020) developed a transgenic zebrafish model with labelled EVs to study osteoblasts. EVs were isolated by flow cytometry and described with spherical morphology by TEM in a size range of 600–2,000 nm, and were characterized as large EVs and apoptotic bodies.

Furthermore, the presence of EVs was confirmed in the serum of Nile tilapia (*Oreochromis niloticus*), Chinese tongue sole (*Cynoglossus semilaevis*), Grass carp (*Ctenopharyngodon idellus*), Atlantic halibut (*Hippoglossus hippoglossus*), Mandarin fish (*Siniperca chuatsi*), Rohu (*Labeo rohita*) and Crucian carp (*Carassius auratus*) (Zhao et al. 2021; Sun et al. 2022, 2017; Zhu et al. 2022; Zhang et al. 2021; He et al. 2021; Tang et al. 2022).

Sun et al (2017) isolated EVs from male and female Chinese tongue sole (*Cynoglossus semilaevis*) fish using centrifugation and precipitation and analyzed the miRNA expression profile of EVs of blood plasma. They described the size of EVs by NTA with a 30–120 nm size range with a spherical shape by TEM. Canonical EV markers CD63, HSP70 and CD81 were identified in the EVs using Western blot.

Zhao et al. (2021) isolated EVs from epidermal mucus of Chinese tongue sole (*Cynoglossus semilaevis*) to analyze the proteomics of EV cargo after *Vibrio harveyi* infection. EVs were isolated using 0.45 μm filtration and Total Exosome Isolation kit. They described the EVs with typical morphology using TEM and with a particle size range of 100 to 400 nm, with 2.40 ± 0.08e + 9 particles/mL concentrations. They also included EV markers HSP90, CD63 and TSG101 in Western blot analysis.

Sun et al. (2022) isolated serum exosomes using ExoQuickTM and ultracentrifugation from Chinese tongue sole (*Cynoglossus semilaevis*) and analyzed the miRNA expression and inflammation in EVs after *Vibrio harveyi* infection. EVs were characterized with a spherical shape with a diameter of 30–295 nm in 3.3E + 9 particles/ml and the canonical EV markers CD63 and CD81 using Western blot.

Zhu et al. (2022) isolated plasma exosomes from Chinese tongue sole (*Cynoglossus semilaevis*) using differential ultracentrifugation. They used 2,000 g for 30 min, 12,000 g for 45 min, 20,000 g for 2 h, 0.22 μm filtration, and ultracentrifuged the samples three times at 120,000 g for 70 min-2 h. EVs were described 50–200 nm in TEM with a spherical and cup shaped morphology and 5.28e + 8—2.71e + 9 particles/mL concentration in nano-flow cytometry. They included CD63, CD81 and HSP70 markers in Western blot analysis and focused on the miRNA cargo of EVs.

Zhang et al. (2021) isolated EVs from grass carp *(Ctenopharyngodon idellus)* kidney (CIK) cells using 300 g for 10 min, 2000 g for 20 min, and 10,000 g for 30 min, 0.22 μm filter, followed by ultracentrifugation at 120,000 g for 70 min to purify the exosomes. The proteomic profile of EVs was investigated after grass carp reovirus (GCRV) infection. They characterized the EVs with cup-shaped bilayer-enclosed morphology using TEM, and with the canonical EV markers CD63, CD81 and TSG101 using Western blot.

He et al. (2021) isolated EVs from Mandarin fish (*Siniperca chuatsi)* serum using centrifugation at 2000 g for 30 min; 12,000 g for 45 min and 110,000 g for 2 h, following a 0.22 µm filtration and from Mandarin fish fry cells (MFF-1 cells) using 300 g for 10 min, 20,000 g for 20 min and 110,000 g for 70 min following a 0.22 µm filtration. They analyzed the anti-viral role of EVs in Infectious spleen and kidney necrosis virus (ISKNV) infection and identified the Mx1 protein in the EV cargo as a key protein and its delivery into recipient cells via EVs. They characterized the EVs with cup-shaped bilayer-enclosed morphology, ranging from 40 to 150 nm in size using TEM, and with the canonical EV markers CD63, TSG101 and HSPA8 using Western blot.

Tang et al. (2022) used 300 g for 10 min, 2000 g for 10 min, 100,000 g for 60 min, 0.22 µm filtration and finally, 120,000 g for 70 min to pellet extracellular vesicles from Rohu (*Labeo rohita*), Crucian carp (*Carassius auratus*) and Nile tilapia (*Oreochromis niloticus*) plasma. They investigated the potential of EVs as biomarkers in ecotoxicology combined with oxidative stress parameters from control and polluted areas. They characterized the EVs with cup-shaped spherical and double layer membrane structure, and their sizes in diameter ranged from 30 and 120 nm and investigated the total protein levels of EVs in the samples, however, they did not include any canonical EV surface membrane protein marker in their study.

Our findings for morphology, size range and particle number of large and small EVs correspond to the expected results according to literature (Table 1). However, it is important to note that the isolation and characterization of extracellular vesicles shows great variability in the literature. There are many available techniques to isolate EVs, for example stepwise centrifugation (Crescitelli et al., 2021), density gradient ultracentrifugation (Karimi et al. 2018), bind-elute and size exclusion chromatography (Corso et al. 2017) and their combination (Onódi et al. 2018) in addition to commercially available kits and many more (Tian et al. 2019). However, the applied pore size for filtering steps may exclude large EVs, and studies often use smaller filter pores (0.1–0.22 µm) and focus their research on small EVs (Crescitelli et al., 2021), despite that the typical size range of the major lipid-bilayer EVs is up to 1000 nm in diameter (Raposo and Stoorvogel, 2013; Davidson et al. 2022). There are also many different methods available to measure the particle number of EVs in the isolates including TEM, NTA, DLS and TRPS, nano-flow cytometry (Anderson et al. 2013), and to analyze the morphology of EVs including TEM, SEM or AFM (Malenica et al. 2021). Indeed, EVs carry a large variety of cargos including miRNA, mRNA, proteins, or lipids (Raposo et al., 2019) which can be subjected for analysis. The large number of available methods of EV isolation and characterization make it difficult to compare the data in the literature (Maas et al. 2015). Studies also use different bodily fluids/medium in highly variable volumes (200–40,000 μl see Table 1) for EV isolation, sometimes from pooled samples of different animals.

Regardless of their biological source or type of EV sub-population, EVs have a specific morphology (Malenica et al. 2021), which is described as a “cup” shape, however the morphology of the EVs show high variability in publications, and most EVs are described rather with a spherical morphology, which can be in relation with many different factors including the sample preparation, method of EV isolation, time of negative staining and whether the EVs were freshly analyzed or stored frozen (Szatanek et al. 2017; Malenica et al. 2021).

The use of canonical EV markers also shows high variability in piscine studies. According to the Minimal Information for Studies of Extracellular Vesicles (2018) (MISEV 2018) guidelines (Théry et al. 2018), surface membrane protein markers, such as tetraspanins (CD9, CD63, CD81) can be used to identify EVs, however MISEV 2018 also states that heat shock proteins (HSPA8, HSPA1A, HSP90AB1), actin (ACT*), tubulin (TUB*), and GAPDH proteins do not qualify as EV-specific components (Théry et al 2018).

Despite the challenges, EV research is an emerging field in many different areas including human medical research and pharmacology (Quadri et al. 2022), and it has started to be introduced to aquatic biology in the past decade (Zhao et al. 2022). Scientific studies are published in several areas, including fish immunology, fish health and welfare or ecotoxicology (Lagos et al. 2017; Iliev et al. 2018; Magnadóttir et al. 2019; Magnadóttir et al. 2020). Since the cargo of EVs depends on the parent cell and reflects its responses to stress (Palviainen et al. 2019), they have the potential to be used as novel and innovative biomarkers in aquaculture. The biogenesis, release and uptake of EVs shed light to new biological processes, and the possibilities of using EVs as biomarkers highlight the significance of their cargo, including protein and miRNA content of the released EVs (Wei et al. 2021).

The isolation, quantity and purity of EV samples are challenging tasks even in the human medical fields, especially from complex bodily fluids without the potential loss of EV subpopulations for characterization (Allelein et al. 2021). In vitro cell line models offer several advantages which can be considered in EV studies, such as they are cost effective, easy and time-effective use, and bypass ethical concerns associated with the use of animals (Soldatow et al. 2013). Cell lines also provide a pure population of cells, which is valuable since it provides a consistent sample and reproducible results and the serum-free medium as an EV source is less complex compared to bodily fluids (Bojmar et al. 2020) resulting in lower levels of contamination and the possibility to investigate specific biological processes of cells.

Here we demonstrated that large and small extracellular vesicles can be successfully isolated using stepwise centrifugation from the medium of rainbow trout liver (RTL-W1), gill (RTgill-W1), intestinal epithelium (RTgutGC) and zebrafish liver (ZFL) cell lines after a 24-h incubation period. To our knowledge this is the first study to characterize the morphology and size range of rainbow trout (*Oncorhynchus mykiss*) and zebrafish (*Danio rerio*) cell line derived large and small EVs using TRPS, DLS and TEM. However, it requires further, extensive studies to assess and describe the cargo of the EVs and how the quantitative and qualitative characteristics of EVs change in different conditions.

## Data Availability

The datasets generated during and/or analysed during the current study are available from the corresponding author on reasonable request.
